# Individual- and community-level social gradients of edentulousness

**DOI:** 10.1186/s12903-015-0020-z

**Published:** 2015-03-11

**Authors:** Kanade Ito, Jun Aida, Tatsuo Yamamoto, Rika Ohtsuka, Miyo Nakade, Kayo Suzuki, Katsunori Kondo, Ken Osaka, for the JAGES group

**Affiliations:** Department of International and Community Oral Health, Tohoku University Graduate School of Dentistry, Sendai City, Miyagi Japan; Division of Oral Health Sciences, Department of Health Sciences, School of Health and Social Services, Saitama Prefectural University, Koshigaya City, Saitama Japan; Department of Dental Sociology, Graduate School of Dentistry, Kanagawa Dental University, Yokosuka City, Kanagawa Japan; Research Team for Human Care, Tokyo Metropolitan Institute of Gerontology, Itabashi Ward, Tokyo Japan; Department of Nutrition, Faculty of Health and Nutrition, Tokaigakuen University, Nagoya City, Aichi Japan; Center for Well-being and Society, Nihon Fukushi University, Nagoya City, Aichi Japan; Department of Policy Studies, Aichi Gakuin University, Nisshin City, Aichi Japan; Center for Preventive Medical Sciences, Chiba University, Chiba City, Chiba Japan

**Keywords:** Dental public health, Edentulous/edentulism, Income inequality, Gender differences, Multilevel analysis

## Abstract

**Background:**

Community-level factors as well as individual-level factors affect individual health. To date, no studies have examined the association between community-level social gradient and edentulousness. The aim of this study was to investigate individual- and community-level social inequalities in edentulousness and to determine any explanatory factors in this association.

**Methods:**

We analyzed the data from the Japan Gerontological Evaluation Study (JAGES). In 2010-2012, 112,123 subjects aged 65 or older responded to the questionnaire survey (response rate = 66.3%). Multilevel logistic regression analysis was applied to determine the association between community-level income and edentulousness after accounting for individual-level income and demographic covariates. Then, we estimated the probability of edentulousness by individual- and community-level incomes after adjusted for covariates.

**Results:**

Of 79,563 valid participants, the prevalence of edentulousness among 39,550 men (49.7%) and 40,013 women (50.3%) were both 13.8%. Living in communities with higher mean incomes and having higher individual-level incomes were significantly associated with a lower risk of edentulousness (odds ratios [ORs] by 10,000 USD increments were 0.37 (95% confidence interval [CI] [0.22-0.63]) for community-level and 0.85 (95% CI [0.84-0.86]) for individual-level income). Individual- and community-level social factors, including density of dental clinics, partially explained the social gradients. However, in the fully adjusted model, both community- and individual-level social gradients of edentulousness remained significant (ORs = 0.43 (95% CI [0.27-0.67]) and 0.90 (95% CI [0.88-0.91]), respectively). One standard deviation changes in community- and individual-level incomes were associated with 0.78 and 0.84 times lower odds of edentulousness, respectively. In addition, compared to men, women living in communities with higher average incomes had a significantly lower risk of edentulousness (p-value for interaction < 0.001).

**Conclusions:**

Individual- and community-level social inequalities in dental health were observed. Public health policies should account for social determinants of oral health when reducing oral health inequalities.

## Background

Severe tooth loss is the 36th most prevalent condition among 291 diseases and it caused a loss of 106 disability-adjusted life-years per 100,000 population [1]. Prevalence of severe tooth loss increases with age. Approximately 20% of the older population experienced severe tooth loss [2]. Severe tooth loss causes chewing difficulties and poor nutritional status [3]. It also affects general health status. For example, tooth loss predicts the onset of future co-morbidities such as dementia [4] and mortality [5].

Recent studies showed that the prevalence of severe tooth loss differed by socioeconomic group [6-8]. These health inequalities are caused by social determinants of health and can be observed on social gradients [9]. Adverse social conditions such as lower income and lower educational attainment affect the health of not only the most disadvantaged people, but also the entire population within a society [10,11]. The differences in social conditions create a stepwise gradient of health conditions between social groups [12,13]. The total loss of teeth (i.e., edentulousness) reflects the social determinants of an individual’s life-course, as it is the result of oral health behavior, oral diseases, and the community health care system [14]. Reducing oral health inequalities is an urgent matter for both researchers and policymakers [15-17]. Furthermore, determining the factors that affect oral health inequalities is important for future public health interventions.

Recent studies have demonstrated that not only individual factors, but also community-level social determinants such as income inequalities or community-level mean income affect the health of individuals and facilitate health inequalities [7,8,18]. Because community factors potentially affect the health of all residents in an area, it is important to understand their effects on health. However, to the best of our knowledge, no study has examined both the individual- and community-level social gradients of edentulousness. Thus, the aims of the present study were: 1) to investigate the association between individual- and community-level incomes and edentulousness, 2) to determine the explanatory factors for edentulousness inequalities, and 3) to investigate gender differences within the socioeconomic inequalities of edentulousness.

## Methods

### Data collection

We used cross-sectional data from the Japan Gerontological Evaluation Study (JAGES) cohort study in Japan. The JAGES project is an ongoing prospective cohort study investigating social and behavioral factors associated with the loss of health related to functional decline or cognitive impairment among individuals aged 65 years or older [6,19,20]. Between August 2010 and January 2012, a total of 169,215 community-dwelling people aged 65 years and older were randomly selected from 31 municipalities in 12 prefectures in Japan and mailed a set of questionnaires. In total, 112,123 people in 31 municipalities participated (response rate = 66.3%). We used data from 79,563 participants without missing responses.

### Outcome variable

The outcome variable for the present analysis was edentulousness (i.e., edentulous or dentulous). Current dental status was measured by a self-administered questionnaire. Respondents were asked “What is the status of your dental health?” with four choices: 1) I have 20 or more natural teeth, 2) I have 10 to 19 natural teeth, 3) I have 1 to 9 natural teeth, or 4) I have no natural teeth. We categorized answers 1-3 as “dentulous” and answer 4 as “edentulous.”

### Main predictors

We used two income variables as the main predictors. The individual-level equivalent household income was obtained and calculated from the questionnaire. The community-level mean income was obtained from national census data [21]. Both income variables were used as continuous variables and the unit used was 10,000 USD (1 USD = 100 JPY).

### Individual-level socio-demographic covariates

Sex, age (65-69, 70-74, 75-79, 80-84 and >84 years old), marital status (currently married, widowed, divorced, never married, and other), and educational attainment (years of school education received (<6, 6-9, 10-12, >12 years, and other)) were used as individual-level socio-demographic covariates. Marital status [22-24] and educational attainment [25,26] were associated with general and oral health status. In addition, both variables in this study were associated with income level. Therefore we included these variables as covariates.

### Community-level socio-demographic covariate

Density of dental clinics is a proxy for access to dental care in communities. A previous study in Japan indicated that density of dental clinics was an appropriate proxy for access to care [27]. Dental status is associated with access to dental care [27]. Density of dental clinics is likely to be higher in urban areas than rural areas [28]. Generally, urban areas are richer than rural areas [29]. Thus, we used density of dental clinics as a covariate of community-level income in this analysis. Density of dental clinics in each municipality in 2010 were obtained from the census data and used as the community-level variable [30].

### Data analysis

In our dataset, 79,563 individuals (individual-level) were nested across 30 municipalities (community-level). We have hypothesized that oral health is affected not only by individual-level socioeconomic status but also by community-level social conditions. To examine the contextual effect of community-level income on edentulousness, we applied a 2-level multilevel logistic regression analysis with random intercepts and fixed slopes. To determine explanatory factors in the association between individual- and community-level incomes and edentulousness, we built the models as follows. Model 1 tested the association between individual- and community-level incomes and edentulousness. Model 2 tested the association between income variables and edentulousness after adjusting for age, sex, and marital status. Model 3 added educational attainment into Model 2. Model 4 was the fully adjusted model, adding the community-level variable (density of dental clinics) into Model 3. To determine gender differences in the effect of both individual- and community-level incomes on dental health, interaction terms were included in the fully adjusted model. To evaluate the degrees of individual- and community-level variances in edentulousness, median odds ratios (ORs) were calculated [31]. To compare the degrees of the association between individual- and community-level income variables and edentulousness, we constructed a fully adjusted model with standardized income variables. When non-standardized income variables were included into the models, they were grand mean centered. Analysis were conducted using MLwiN version 2.28 (Centre for Multilevel Modelling, University of Bristol, UK).

### Ethical considerations

Ethical approval for the study was obtained from the Ethics Committee at Nihon Fukushi University, Japan (Approval number: 10-05).

## Results

The average ages of 39,550 men (49.7%) and 40,013 women (50.3%) were 73.5 (SD = 5.97) and 73.7 (SD = 6.17) years old, respectively. The prevalence of edentulousness was 13.8% for both men and women. Table 1 shows the demographic distribution of the variables by dental status. Edentulous individuals had significantly lower incomes and lived in communities with lower mean incomes (p < 0.001).

**Table 1:** **The demographic distribution of variables by dental status (n = 79,563)**

***Categorical variables***		**Dentulousness n (%)**	**Edentulousness n (%)**	**p-value**
Sex	Male	34,083 (86.2)	5,467 (13.8)	0.798^†^
	Female	34,507 (86.2)	5,506 (13.8)	
Age	65-69 ys	23,239 (94.6)	1,327 (5.4)	p < 0.001^†^
	70-74 ys	21,560 (90.3)	2,314 (9.7)	
	75-79 ys	14,212 (83.2)	2,877 (16.8)	
	80-84 ys	6,899 (72.8)	2.573 (27.2)	
	>84 ys	2,680 (58.7)	1,882 (41.3)	
Marital status	Married	52,769 (88.1)	7,115 (11.9)	p < 0.001^†^
	Widowed	12,185 (78.6)	3,311 (21.4)	
	Divorced	2,007 (86.7)	307 (13.3)	
	Never married	1,316 (88.4)	173 (11.6)	
	Other	313 (82.4)	67 (17.6)	
Educational attainment	<6 ys	1,120 (61.70	694 (38.3)	p < 0.001^†^
	6-9 ys	27,979 (82.7)	5,853 (17.3)	
	10-12 ys	25,428 (89.4)	3,023 (10.6)	
	>12 ys	13,650 (91.3)	1,299 (8.7)	
	Other	413 (79.9)	104 (20.1)	
***Continuous variables***		Mean (SE)		
Density of dental clinics (per 10 thousand population)		4.45 (±0.837)	4.31 (±0.699)	p < 0.001^‡^
Individual income (10 thousand US dollars*)		2.39 (±1.553)	1.95 (±1.467)	p < 0.001^‡^
Community income (10 thousand dollars*)		3.18 (±0.297)	3.09 (±0.285)	p < 0.001^‡^

^†^p-value for chi-squared test.

^‡^p-value for *t*-test.

*1 US dollar = 100 Japanese Yen.

Table 2 shows the results of the multivariate multilevel analysis. In the intercept-only model (not shown), there was a significant difference in edentulousness between municipalities (community-level variance: Ωγ = 0.262, SE = 0.069). The median OR in the model was 1.629, which indicated that if a person moved to another municipality with a higher probability of poor dental status, their median risk of edentulousness would increase 1.629 times.

**Table 2:** **Association of edentulousness with individual- and community-level variables determined by multilevel logistic regression (n = 79,563)**

	**Model 1**	**Model 2**	**Model 3**	**Model 4**	**Model 5**	**Model 6**
	**OR (95% CI)**	**OR (95% CI)**	**OR (95% CI)**	**OR (95% CI)**	**OR (95% CI)**	**OR (95% CI)**
**Fixed effect**						
***Individual-variables***						
Individual income (10 thousand US dollars)	0.85 (0.84-0.86)	0.87 (0.86-0.88)	0.90 (0.88-0.91)	0.90 (0.88-0.91)	0.90 (0.88-0.92)	0.90 (0.88-0.91)
Educational attainment (ref:>12 ys)			1.00	1.00	1.00	1.00
<6 ys			2.19 (1.94-2.47)	2.19 (1.94-2.47)	2.19 (1.94-2.47)	2.19 (1.93-2.48)
6-9 ys			1.61 (1.50-1.73)	1.61 (1.51-1.73)	1.62 (1.51-1.73)	1.62 (1.51-1.73)
10-12 ys			1.15 (1.07-1.24)	1.15 (1.07-1.24)	1.16 (1.08-1.24)	1.16 (1.08-1.25)
Other			1.79 (1.41-2.28)	1.80 (1.42-2.28)	1.80 (1.42-2.28)	1.80 (1.42-2.29)
***Community-variables***						
Community income (10 thousand US dollars)	0.37 (0.22-0.63)	0.39 (0.25-0.61)	0.41 (0.27-0.63)	0.43 (0.27-0.67)	0.43 (0.27-0.67)	0.53 (0.33-0.85)
Density of dental clinics (per 10 thousand population)				0.96 (0.78-1.19)	0.96 (0.78-1.19)	0.96 (0.78-1.18)
Interaction term (Sex*Individual income)					0.98 (0.95-1.02)	
Interaction term (Sex*Community income)						0.63 (0.54-0.73)
Random effects (SE)	0.148 (0.039)	0.105 (0.028)	0.095 (0.026)	0.095 (0.026)	0.095 (0.026)	0.095 (0.026)
Median OR	1.443	1.362	1.342	1.342	1.342	1.342

Model 1: Adjusted for individual- and community-level incomes.

Model 2: Model 1 + age, sex, and marital status.

Model 3: Model 2 + educational attainment.

Model 4 (full model): Model 3 + community-variable (density of dental clinics).

Model 5,6: Model 4 + each interaction term.

*1 US dollar = 100 Japanese Yen.

**Figure 1:**
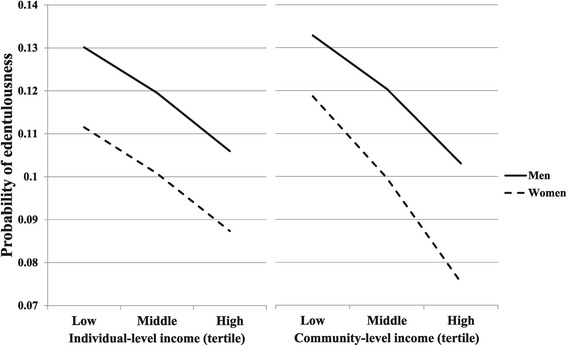
**Gender difference in the association between individual- and community-level incomes and probability of edentulousness.** Compared to men, women living in areas with higher community-level incomes had a lower probability of edentulousness.

Univariate analyses showed that both individual- and community-level incomes were associated with lower risk for edentulousness; ORs of individual- and community-level incomes were 0.84 (95% confidential interval [CI] [0.84-0.86]) and 0.33 (95% CI [0.19-0.60]), respectively. Then, we included both income variables into the same model. Having a 10,000 USD higher income and living in a community with a 10,000 USD higher mean income were associated with 0.85 times and 0.37 times lower risk for edentulousness, respectively (Model 1). Individual characteristics mediated these relationships by 13.5% (individual-level income) and 3.4% (community-level income), respectively (Model 2, calculated from the ORs [32]). Educational attainment further attenuated the ORs of individual- and community-level income variables by 20.5% and 3.8%, respectively (Model 3). The community-level covariate, density of dental clinics, only mediated the association between community-level income and edentulousness (2.1% reduction of the OR, Model 4). Even after considering all covariates, there remained significant geographical differences and individual- and community-level social gradients for edentulousness (Model 4). When standardized income variables were included in Model 4 instead of non-standardized income variables, ORs for individual- and community-level income variables were 0.84 (95% CI [0.82-0.87]) and 0.78 (95% CI [0.68-0.89]), respectively. There was a significant interaction between gender and community-level income, although the interaction between gender and individual-level income was non-significant (Models 5 and 6). Compared to men, women living in areas with higher community-level incomes had a lower probability of edentulousness (Figure 1). For individual-level income, similar social gradients were observed among both men and women (Figure 1).

## Discussion

To the best of our knowledge, this study was the first to examine both the individual- and community-level social gradients of edentulousness using a multilevel analysis. This large-scale multilevel analysis demonstrated that not only individual-level income but also community-level income showed social gradients for edentulousness. Present study also investigated the factors which associated between income inequalities and dental status. Individual socioeconomic characteristic partially mediated the association between both individual- and community-level incomes and edentulousness. The community’s socio-demographic characteristic also partially mediated the association between community-level income and edentulousness. However, even after adjusting for all covariates, individual- and community-level social gradients remained. In addition, compared to men, women living in municipalities with higher community-level incomes derived greater oral health benefits from the social environment.

The present study reports similar result to previous studies using non-oral health outcomes, which have suggested that community-level social factors affect population health [18]. Those systematic review and meta-analysis showed that poor community-level socioeconomic environment increased resident mortality [18]. A meta-analysis of 11 studies with smaller sample sizes indicated that living in areas with low socioeconomic status increased mortality 1.11 times compared to areas with high socioeconomic status [18]. Another meta-analysis of seven studies with larger sample sizes also demonstrated that living in low socioeconomic status areas increased mortality 1.07 times [18]. In oral health studies, regardless of individual-level income, adults living in affluent areas had a higher number of remaining teeth than those living in deprived areas, after adjusting for age, sex, and educational attainment [8]. Dental health is considered to be affected to a greater extent by community-level factors. Previous studies conducted in one Japanese prefecture reported larger geographical differences in the dental outcome of number of remaining teeth compared to self-rated health [7]. Therefore, a public health intervention considering community-level social determinants would be more effective.

There are at least three possible pathways between community-level income and oral health. First, access to dental care could explain the mechanism. Second, individual health behaviors are formed by the surrounding environment. Third, people living in affluent communities are less likely to have psychosocial stress than those living in deprived communities. In relation to the first pathway, although we considered access to dental clinics in the models, there might be unexplained variance of the outcome associated with access to dental care. A previous study demonstrated that low-income individuals had less access to dental clinics than high-income individuals [33]. Moreover, access to dental clinics was significantly associated with area-level income after adjusting individual income [28]. This study suggested that people living in affluent areas were more likely to visit a dental clinic than those in deprived areas, regardless of individual socioeconomic status. Although the variable we used, density of dental clinics, could change throughout the life-course of each respondent, we could not consider possible changes in this variable. Therefore, this might have caused the unexplained variance of the outcome, which was associated with access to dental care. For the second pathway, compared to deprived communities, affluent communities tend to have positive social environments, including sufficient grocery stores with fresh and healthy food, public safety, and good access to hospitals and dental clinics [34,35]. People living in affluent communities tend to eat more fruits and sugar-free foods because they can easily purchase healthy foods at grocery stores in their communities [36,37]. In addition, people living in affluent communities are also more likely to drink healthy beverages, such as non-sugared teas rather than sodas [38]. Sugar is an established risk of dental caries [39]. Moreover, recent study also indicated that sugar associated with risk of periodontal diseases [40]. Healthy lifestyles can help prevent them. For the third pathway, people living in affluent communities are less likely to have psychosocial stress because of increased safety, good social capital (e.g., social connections and social networks), and social norms than those living in deprived communities [41]. Psychosocial stress is also associated with smoking status, which affects periodontal diseases causing tooth loss [42]. In addition, community public safety affects oral health by reducing the possibility of dental injuries. Dental injury was affected by community social environment [43].

Present study showed that community explanatory variable partially mediated the association between community-level income and edentulousness. To examine the possibility of the pathway “access to dental care”, we include the variable into the model. However, variable on access to dental care explained only 2.1% of the association between community-level income and edentulousness. Further studies that consider the wider range of variables related to the pathway, such as social capital and geographical clustering of dental health behaviors, are needed.

In the present study, women’s dental health was affected by community-level income to a greater extent than men’s health. Previous studies on other health outcomes have reported similar results. Compared to men, the self-rated health of women is considered to be affected to a greater extent by the neighborhood social environment [44]. Another study on self-rated health reported similar findings and the authors suggested that this might be because women tend to spend more time at home and in the community [45]. Thus, women were more likely than men to communicate with neighbors. Therefore, women’s health behaviors are more likely to be affected by neighbors though informal social control and social influence. A previous study in Japan demonstrated that, for older women, the distance to a dental clinic was an important factor for dental attendance, while distance was not significantly associated with access to dental care among older men [27]. Because many older women in Japan do not have a driver’s license, public transportation is considered an important factor for dental clinic access [27].

### Public health implications

Community factors are important because they potentially affect the health of all residents in a given area. The present study revealed the importance of community-level socioeconomic status on oral health. Therefore, interventions should focus not only on individual efforts but also consider community-level social determinants underlying the oral health of a population. Therefore, after relevant factors are determined by future studies, *upstream* approaches including structural and environmental interventions for improving various social determinants of communities (e.g., smoking policies for public spaces, food policies for reducing sugar consumption, health care system reforms for improving access to preventive and curative care, and access to fluoride in the water system or in schools) are necessary for reducing oral health inequalities [46-48]. In addition to these *upstream* approaches, building society which focuses on not only economic growth, but also fair distribution of well-being of individuals are required [34]. As various socioeconomic environment of community affect health of residents, broader social and economic policies should consider health and well-being of residents [34].

### Limitation and strengths

This study has some limitations. First, this was a cross-sectional study; thus, we cannot rule out the possibility of reverse causation. Consequently, prospective follow-up studies are required. Second, though the validity of self-reporting number of remaining teeth was validated, measurements were obtained from a self-administered questionnaire [49]. If we can obtain clinical measurements of remaining teeth, which are more accurate than self-administered questionnaires, the association between income variables and edentulousness will be strengthened. Third, there might have a potential bias because of a lack of many cases. The main strength of this study was its large sample size. In addition, our survey was conducted across an adequate number of municipalities with various characteristics and we used appropriate statistical analysis. Therefore, the present study could legitimately describe the effects of community factors.

## Conclusion

In conclusion, community-level income, as well as individual-level income, formed social gradients for edentulousness, even after accounting for individual- and community-level factors. The oral health of women living in municipalities with higher community-level incomes benefited from the social environment.

## Availability of supporting data

Raw data is available from corresponding author.

### Standards of reporting

This study was prepared according to STROBE check list for cross-sectional studies.

### Consent

Present study was an observational study and not using human biological specimens. We explained all relevant details regarding the study to be carried out and provide each prospective subject an opportunity to refuse inclusion in the research. Subject who consented to participate in the study wrote the self-reported questionnaire and send it by mail.

## Abbreviations

JAGES: Japan Gerontological Evaluation Study
OR: Odds ratio
CI: Confidence interval
